# Development of a theoretical construct for teacher expertise in the Chinese context and identification of its components: A mixed-methods study

**DOI:** 10.3389/fpsyg.2023.1121109

**Published:** 2023-02-10

**Authors:** Yonghong Cai, Yanli Li, Runjia Tang

**Affiliations:** Faculty of Education, Beijing Normal University, Beijing, China

**Keywords:** theoretical construct, construct components, teacher expertise, expert teachers, mixed-methods study

## Abstract

**Introduction:**

Teacher expertise is a vital element of teaching quality. Examining what constitutes teacher expertise has important implications for the theoretical development and practical application of teacher expertise. The present study was conducted to develop a theoretical construct for teacher expertise in the Chinese context, identify its components, and verify the validity of this construct.

**Methods:**

This study adopted an exploratory sequential mixed-methods design. To develop a construct for teacher expertise and identify its components, critical incident interviews were conducted with 102 primary and secondary school teachers. Grounded theory analysis was applied to code 621 stories from critical incident interviews. To verify the construct validity and discriminant validity, a survey of 1,041 teachers was conducted in 21 primary schools and 20 secondary schools in Hebei and Shanxi provinces. Confirmative factor analysis, Kruskal-Wallis test, and Mann-Whitney test were used to evaluate the validity of the construct.

**Results:**

Knowledge structure, teaching ability, and professional development agency constituted the construct for teacher expertise. This construct had good construct validity and discriminant validity. Knowledge structure could not identify expertise. Teaching ability and professional development agency could differentiate between expert and non-expert teachers.

**Discussion:**

Teacher expertise is a complex, multidimensional, and adaptive construct. The construct can be used as a valid and reliable instrument to identify and develop teacher expertise. Moreover, this study expands on prior studies and complements recent theoretical models describing teacher expertise.

## 1. Introduction

The teacher’s level of expertise has a strong influence on student learning (Bond et al., 2000). Examining what constitutes teacher expertise has important implications for understanding what it takes for a novice to become an expert in the field. A novice and an expert can be distinguished from each other by qualitative differences in the components of their expertise (Moon et al., 2013).

The past few decades have seen the development of a substantial body of literature on teacher expertise (Berliner, 2001; Hammerness, 2004). Many of the early studies of routine expertise compared novice and expert teachers. Being an expert teacher is commonly acknowledged to involve a strong knowledge of teaching and effective teaching practices (Lee and Yuan, 2021). Expert teachers have a richer, more sophisticated, and more elaborate knowledge base; more developed schemata or routines (Borko and Livingston, 1989; Westerman, 1991); and greater automaticity. They are more adaptable and flexible in their teaching, more sensitive to task demands and social situations, and faster and more accurate in recognizing patterns and typical events (Berliner, 2004; Wolff et al., 2015) than non-experts. In contrast, the latest studies on adaptive expertise have turned their attention to the dynamic and multifaceted nature of teacher expertise. Teacher expertise is regarded as a complicated construct because of its context-dependent and non-linear nature (Sternberg and Horvath, 1995; Berliner, 2001; Tsui, 2009; Collins and Evans, 2018). From this perspective, an expert teacher needs not only disciplinary knowledge and skill but also competence in responding adaptively and innovatively to the exigencies of teaching and learning through flexible and creative strategies.

Literature on the components of teacher expertise is still lacking in consistency and agreement on many features. The debate concerning the components of teacher expertise mainly focuses on whether it is a unidimensional competence or a multidimensional one containing both cognitive ability and motives. The unidimensional view holds that teacher expertise involves only cognitive competence in either knowledge or experience or deliberate practice (Smith and Strahan, 2004). This perspective oversimplifies teaching situations and neglects non-cognitive components such as emotional management, motivation, and confidence. The bidimensional view holds that teacher expertise includes objective and subjective expertise (Germain, 2006) or cognitive and non-cognitive components (Van der Heijden, 2003). The tridimensional view holds that teacher expertise includes cognitive, non-cognitive, and contextual components (Shanteau, 1992), such as professional knowledge, experience, and deliberate training (Herling, 2000). Recently, teacher expertise has generally been regarded as a knowledge-based comprehensive competence in dealing with complex teaching situations (Germain and Tejeda, 2009), emphasizing components such as innovation, motivation, enthusiasm, beliefs, and personality (Hattie, 2012; Eaude, 2014; Anthony et al., 2015). These disputes have constrained the theoretical development of teacher expertise and its application in teachers’ professional development (Shanteau, 1992; Germain and Tejeda, 2012). Moreover, as teaching is contextually situated, developing a set of objective criteria that can be applied across all contexts and cultures is difficult (Leinhardt, 1990; Turner-Bisset, 2013). Certain dimensions of teacher expertise may be culturally specific (Ferrari, 2001; Mieg, 2001). Therefore, it is necessary to further explore the components of teacher expertise in different cultural contexts.

The goal of this study is thus to develop a theoretical construct for teacher expertise and identify its components in the Chinese context. The findings of this study contribute to a better understanding of how to develop a construct for teacher expertise and its components in the Chinese context. First, we develop a construct for teacher expertise and its components using qualitative research methods of critical incident interviews and grounded theory methods. The qualitative results extend the previous study on routine and adaptive expertise. Besides the cognitive and affective components of teacher expertise, the results identify the role of agency in developing teacher expertise. The virtue of morality is an important component of teacher expertise in the Chinese context. Second, we verify the construct validity and discriminant validity of teacher expertise using quantitative research methods. Professional development agency is proved to be the most important component differentiating expert teachers from experienced or novice ones. We provide a reliable tool for identifying teacher expertise and helping teachers acquire expertise.

## 2. Theoretical framework

### 2.1. The nature of teacher expertise

Teacher expertise can be understood as a set of individual characteristics that are causally related to excellent performance by teachers. It is characterized by long hours of hard work in which the expert teacher is engaged in reflection and conscious deliberation; problematizing the unproblematic; and maximizing opportunities afforded by the context to extend their problem-solving competence. Early studies (Turner-Bisset, 1999; Berliner, 2001) compared expert and novice performance. More recent studies have investigated teacher expertise from a developmental perspective and have understood expert knowledge as constituted by the teacher’s participation in the social practice of teaching. Expertise has come to be understood as a *process* rather than a *state* (Bereiter and Scardamalia, 1993), and the development of expertise over time has been explored (Bullough and Baughman, 1995, 1997; Tsui, 2003). These two perspectives have yielded quite different descriptions of teacher expertise (see Table 1).

**TABLE 1:** Comparison of routine expertise and adaptive expertise.

Elements to be compared	Routine expertise	Adaptive expertise
Nature	Longitudinal competence in similar situations	Horizontal competence in different situations
Perspective	Individual/Cognitive psychology	Individual/Cultural–cognitive psychology
Assumption	Expertise originates from cognitive accumulation	Expertise originates from cognitive conflict
Research significance	Transfer of expertise	Creation of expertise
Characteristics	Speed, accuracy, and automaticity	Flexibility, adaptivity, and creativity
Components	Declarative and procedural knowledge	Conceptual knowledge; explanation of meaning
Expertise development	Observation and imitation; experience and repeated practice under controllable conditions	Profound understanding of conceptual knowledge; exploration of and adaptation to changeable situations; studying with the explicit aim and intention of pursuing self-improvement

#### 2.1.1. Expertise as a state: Routine expertise

The early studies of teacher expertise mostly took the form of comparisons between experts and novices. In these studies, teacher expertise was seen as a static state reached after years of teaching experience. Dreyfus and Dreyfus (1986) proposed a five-stage model of skill acquisition from novice to expert: novice, advanced beginner, competent, proficient, and expert stages. Each stage has a minimum threshold with fixed criteria, and anyone who meets or surpasses that threshold can be identified as an expert. The five-stage model identifies the necessity of going through stages to reach expertise in teaching. This can be partially attributed to the repertoire of pedagogical techniques and skills that expert teachers have developed through years of teaching practice (Turner-Bisset, 1999; Berliner, 2001). The development of routine expertise relies on deliberate practice or the accumulation of experience. Studies of expertise have highlighted the importance for experts of a rich and integrated knowledge base and of the use of such a knowledge base to solve familiar problems more quickly and accurately (Berliner, 1994; Sternberg and Horvath, 1995). However, these studies have been criticized for not being able to reflect teachers’ work accurately because experience is often mistaken for expertise (Bereiter and Scardamalia, 1993). One can never be sure whether the characteristics identified in these studies are critical features of expert performance or merely indications of experienced performance.

#### 2.1.2. Expertise as a process: Adaptive expertise

Expertise has increasingly come to be viewed not as a state or as the acquisition of skills, but rather as a complex construct of adaptations of mind and body to task environments in service of representative task goals and activities, which include substantial self-monitoring and control mechanisms.

Adaptive expertise is a broad structure encompassing a range of cognitive, motivation, identity, or personality components, habits of mind, and dispositions (Hatano and Oura, 2003; Crawford et al., 2005). To become more situationally adaptive, teacher expertise needs to be stimulated by innovative situations, and there needs to be space for the role played by the teacher’s agency (Hatano and Inagaki, 1986). Experts seem to be able to better understand the requirements of the situation and to keep enhancing their competence by setting very high standards for themselves and working very hard to reach those standards. They restructure, reorganize, and refine their representation of knowledge and procedures for efficient application to their work environments (Bereiter and Scardamalia, 1993; Ericsson and Lehmann, 1996). Therefore, adaptive expertise is set apart through competencies such as flexibility, innovation, continuous learning, seeking out challenges, and creativity (Barnett and Koslowski, 2002; Hatano and Oura, 2003; Crawford et al., 2005; Martin et al., 2006; Mylopoulos and Scardamalia, 2008; Varpio et al., 2009). The renewal of knowledge and experience is vital to developing and maintaining expertise (Tsui, 2003). The focus on “change,” “development,” and the “social environment” distinguishes research on adaptive expertise from research on expert performance (Ericsson, 2007; Ericsson and Towne, 2010). Process accounts can help explain varying degrees of competence among experts.

These two perspectives on expertise are not necessarily in conflict with each other (Wineburg, 1998), but rather represent two different aspects of expertise. When experts work in their own specific domains, their expertise is characterized by efficiency, fluidity, and effortlessness. When they work in new areas outside their specific domains, they are capable of adapting their expert knowledge to the new situation and solving the problem at a deeper level. The former type of expertise has been described as “routine” and the latter as “adaptive” (Hatano and Inagaki, 1986; Berliner, 2001). Therefore, routine expertise should be taken as part of adaptive expertise, and experts should both identify the components of routine expertise, such as domain knowledge, experience, and problem-solving skill, and emphasize the components of adaptive expertise, such as initiative, flexibility, and interpersonal interaction.

### 2.2. The components of teacher expertise

In accordance with these views of expertise as “routine” or “adaptive,” there are two main perspectives on what constitutes teacher expertise. One is that it is constituted by cognitive capacity, involving professional knowledge and competence, experience, or deliberate practice (Shanteau, 1992; Herling, 2000; Smith and Strahan, 2004), and the other is that it is constituted by multiple capacities, involving cognitive and non-cognitive components of innovation, such as motivation, beliefs, emotional management, and affective attributes (Van der Heijden, 2003; Hattie, 2012; Eaude, 2014; Anthony et al., 2015).

The cognitive approach has dominated the field of expertise studies. Cognitive research has typically explained performance excellence in terms of either an expert’s knowledge base or information-processing skills. This old formula of “knowledge, skill, and disposition” seems too static and individualistic, limiting both the understanding of what teachers need to know, are able to do, and care about and how to ensure they develop their capacities over time. In more recent studies, the sociocultural view has been gaining increasing attention (Greeno et al., 1996). This view emphasizes the notion that the acquisition of expertise is a social process (Lave and Wenger, 1991; Wenger, 1999). Teaching is increasingly understood as a continuously evolving activity and as a socially mediated practice. Classrooms and schools are dynamic environments that change according to the pupils, curriculum, and social environment. Consequently, teachers’ capacities must be constantly developing and changing, which suggests that adaptability is a critical dimension. Adaptive expertise (Zellermayer and Margolin, 2005) is constituted by a personalized, practice-oriented, metacognitive, and context-specific network of knowledge, beliefs, and values (Smith, 2005). From this point of view, teacher expertise lies in establishing routines to deal with repetitive work while being prepared to adapt to specific circumstances that change rapidly and often unpredictably. Table 2 presents different perspectives on the components of teacher expertise, including both routine expertise and adaptive expertise.

**TABLE 2:** Studies on the components of teacher expertise.

Researcher	Cognitive components	Non-cognitive components
Hatano and Inagaki (1986)	Procedural knowledge, conceptual knowledge, procedural skills	/
Berliner (1990)	Contextualized knowledge, fast and accurate pattern-recognition capabilities, use of knowledge, extensive pedagogical content knowledge, better problem-solving strategies, sensitivity to task demands and social situations	Opportunistic and flexible in teaching, adaptation, and modification of goals for diverse learners
Bereiter and Scardamalia (1993)	Formal or book knowledge, informal knowledge or common sense, impressionistic knowledge or intuition, self-regulatory knowledge, progressive problem-solving	Metacognitive controls that facilitate use of the forms of knowledge, willingness to tackle challenging problems that increase expertise
Shanteau (1992)	Domain knowledge, cognitive characteristics, cognitive skills, task characteristics, decision-making strategies	/
Sternberg and Horvath (1995)	Domain knowledge, efficiency of problem-solving, insight	/
Herling (2000)	Knowledge, experience, problem-solving	/
Berliner (2001)	Use of knowledge, extensive pedagogical content knowledge, problem-solving strategies, adaptation and modification of goals, decision-making, monitoring of learning and providing feedback to students, respect for students	Challenging objectives, sensitivity to context, passion for teaching, motivation, self-efficacy
Barnett and Koslowski (2002)	Knowledge, experience, problem-solving performance, theory-based reasoning	Motivation
Hatano and Oura (2003)	Rich and well-structured domain knowledge, years of experience, peer assistance	Confidence, flexible and innovative competence, exploration and reflection, creativity
Berliner (2004)	Years of experience, peer coaching, domain-specific knowledge and skills, fast and accurate pattern-recognition capabilities, rich personal sources of information	Opportunistic and flexible competence, sensitivity to task demands, and social situations
Ericsson (2006)	Experience, deliberate practice, talent, high-quality instruction, task knowledge, performance skills, goal setting, strategic planning	Self-motivational beliefs, forethought, self-reflection, performance control, outcome expectations, task value, goal orientation, metacognitive self-monitoring, self-evaluation and judgment
De Arment et al. (2013)	Cognitive skills	Metacognitive awareness, dispositional characteristics such as curiosity, innate motivation, enjoying challenges, willingness to change, taking managed risks, feedback
Carbonell et al. (2014)	Domain-specific knowledge and skills, Domain-independent skills, past experience	Self-efficacy, emotional regulation, agreeableness, conscientiousness, extraversion, emotional stability/neuroticism, openness to experience
Anthony et al. (2015)	Pedagogical knowledge, interactions, responsibilities	Concern, control, curiosity, confidence, commitment, efficacy/agency
Männikkö and Husu (2019)	/	Reflection process and personal practice
Raduan and Na (2020)	Experience, competence (knowledge, skills, attitude)	Commitment, efficacy, emotional involvement, interest, situational awareness

### 2.3. The present study

There may be cultural differences in terms of perceptions of what constitutes teacher expertise in teaching. Alexander (2000) documented vast differences in what is considered acceptable for teaching in five countries. To date, no commonly accepted criteria for identifying expert teachers have been established. However, the study of cultural differences in teacher expertise remains a largely unexplored territory in the teacher education literature. In Japanese culture, close interpersonal relationships are considered a prerequisite for teaching and learning. Developing interpersonal relationships with students is more important than developing teaching competence (Shimahara and Sakai, 2018). In Chinese culture, expert teachers are defined not only in terms of their commitment to students but also their commitment to the subject of teaching (Ma, 2010). The components of teacher expertise overlap with teachers’ professional quality, which is constituted by professional knowledge, professional competence, and professional ideas and ethics. Professional knowledge refers to pedagogical knowledge, subject knowledge, subject teaching knowledge, and general knowledge. Professional competence refers to teaching skills, classroom management ability, interpersonal relationships, and self-reflection and development. Studies on the characteristics of expert teachers in the Chinese context are summarized in Table 3. Most of these studies are based on routine expertise, in which teachers’ professional growth is divided into different phases, such as novice, proficient, and expert levels. However, no evidence proves that these characteristics can differentiate between experts and novices or the merely proficient. Whether teachers’ professional development from novice to expert follows a linear or non-linear path is also unclear. How a teacher grows from novice to expert is also worthy of further study. In this study, we use mixed-methods research design to develop a theoretical construct for teacher expertise in the Chinese context, identify its components, and verify the validity of this construct. We address the following research questions: (1) What is the construct for teacher expertise and its components? (2) Whether the construct for teacher expertise is valid and can differentiate between expert and non-expert teachers?

**TABLE 3:** Studies on the characteristics of expert teachers in the Chinese context.

Researcher	Components
Ye (1998)	Knowledge structure, teaching capacity, professional ideas
Lian (2004)	Teaching strategy, personality, motivation, occupational psychology
Li and Huang (2004)	Professional knowledge, problem-solving skills, ways of thinking, beliefs, motivations
Wang (2011)	Understanding of concepts, flexible responses to situations, learning preferences, meta-cognition
Li and Kaiser (2011)	Having sound subject content knowledge of teaching topics, appropriately identifying and dealing with difficult content points in students’ learning, emphasizing the development of students’ mathematical thinking and ability, using mathematics problem-solving and posing to develop effective classroom instruction, emphasizing and practicing student-centered instruction, motivating students
Yang (2013)	Broad and profound knowledge base, teaching with flexibility, teaching with coherence, teaching with balance, teaching with the aim of promoting students’ higher-order thinking skills, contemporary–constructivist-oriented beliefs, consistent relationship between beliefs and practices, reflection on teaching

## 3. Materials and methods

This study adopted an exploratory sequential mixed-methods design (Creswell and Creswell, 2018), where the quantitative phase of data collection and analysis follows the qualitative phase of data collection and analysis (Fetters et al., 2013). This mixed approach has the advantage of collecting, analyzing, and combining both qualitative and quantitative data during the research process to capture both rich qualitative descriptions and quantitative data. Adopting a mixed-methods approach can provide more enhanced and comprehensive answers to research questions (Creswell and Clark, 2017). The qualitative method provided the answer to the first research question, whereas the quantitative method provided the answer to the second question. For the qualitative method, we used critical incident interviews and grounded theory to conceptualize the construct for teacher expertise, identify its components, and develop the initial instrument of teacher expertise. Rich, detailed data were collected from the teachers’ stories of critical incidents. Grounded theory was used to identify common themes. For the quantitative methods, we used confirmatory factor analysis, Kruskal-Wallis test, and Mann-Whitney test to verify the validity of the construct for teacher expertise. In moving from qualitative analysis to developing a survey scale, the codes become variables, themes become scales, and the quotations become survey items. The quantitative results further test the qualitative results with a large-scale survey and complex statistical analyses. Thus, the exploratory mixed methods study design increases the validity and reliability of the instrument of teacher expertise.

### 3.1. Participants

In the qualitative phase, 102 teachers from Beijing primary and secondary schools were interviewed using critical incident interviews. The participants’ specific information, such as their gender, teaching experience, school level, subject taught, and professional title, was considered to ensure the representativeness of the sample. Table 4 represents the sample of teacher participants. The age range of the participant teachers was 23–42 (Mean = 32.64, SD = 6.89). Of the teacher participants, 32 (31.4%) were male and 70 (68.6%) were female; 20 (19.6%) had 0–5 years of teaching experience, 45 (44.1%) had 5–10 years of teaching experience, 27 (26.5%) had 11–15 years of teaching experience and 15 (9.8%) had over 15 years of teaching experience; 43 (42.2%) taught at elementary level and 59 (57.8%) taught at secondary level; 77 (75.5%) taught social science subjects, 25 (24.5%) taught science subjects; 41 (40.2%) had a middle-level title, and 22 (21.6%) had a senior-level title.

**TABLE 4:** Teacher participants (*N* = 102) in the qualitative study.

Category	Representation
Gender	Male: 32 (31.4%) Female: 70 (68.6%)
Teaching experience	< 5: 20 (19.6%) 6–10: 45 (44.1%) 11–15: 27 (26.5%) > 15:10 (9.8%)
School level	Elementary: 43 (42.2%) Secondary: 59 (57.8%)
Subject taught	Science: 25 (24.5%) Social Science: 77 (75.5%)
Professional title	Middle level: 41 (40.2%)
	Senior level: 22 (21.6%)

In the quantitative phase, a questionnaire survey was conducted in 21 primary schools and 20 secondary schools in Hebei and Shanxi provinces. Stratified sampling and cluster random sampling were used in the survey. A sample of 1,200 teachers was approached, and 1,041 valid questionnaires were returned for an effective recovery rate of 86.8%. The age range of the participant teachers was 20–61 (Mean = 35.60, SD = 7.94). Table 5 represents the sample of teacher participants. Of the 1,041 valid samples, 85.6%). 195 (18.7%) had 0–3 years of teaching experience, 206 (19.8%) had 4–10 years of teaching experience, 235 (22.6%) had 11–17 years of teaching experience, and 405 (38.9%) had over 18 years of teaching experience; 172 (16.5%) had a junior college degree, 771 (74.1%) had a bachelor’s degree, and 38 (3.7%) had a master’s degree or above; 351 (33.7%) had a middle-level title, 690 (66.3%) had a senior-level title or above; 241 (23.2%) had received a district-level honor or above, 192 (18.4%) had received a school-level honor, and 608 (58.4%) had not received any honor.

**TABLE 5:** Teacher participants (*N* = 1,041) in the quantitative study.

Category	Representation
Gender	Male: 143 (13.7%) Female: 898 (86.3%)
Teaching experience	< 3: 195 (18.7%) 4–10: 206 (19.8%) 11–17: 235 (22.6%) > 18:405 (38.9%)
Educational level	Junior college: 172 (16.5%) Bachelor’s degree: 771 (74.1%) Master’s degree or above: 38 (3.7%)
Professional title	Middle level: 351 (33.7%) Senior level: 690 (66.3%)
Honor	District level: 241 (23.2%) School level: 192 (18.4%) No honor: 608 (58.4%)

### 3.2. Instruments and procedures

#### 3.2.1. Critical incident interviews

In the qualitative phase, the participants were asked to share stories about critical incidents with which they had dealt in the course of their teaching and to account for how they thought about and acted upon these incidents. General guidelines for the form and content of the stories were provided to obtain more helpful information. Specifically, the participants were asked to respond to stories describing: (a) the circumstances of three successful incidents, including the context, people involved, cause and effect, results, and especially their judgment, self-evaluation, and any countermeasures taken in the incidents; (b) the circumstances of three unsuccessful incidents, including the context, people involved, cause and effect, results, and especially their judgment, self-evaluation, and any countermeasures taken in the incidents; and (c) the qualities and conditions necessary to become an expert, or the characteristics of excellent teachers. The interviews were audio-recorded to ensure accuracy in recording the responses. The interviews were transcribed verbatim and processed as text.

#### 3.2.2. Teacher expertise survey

In the quantitative phase, the study developed a teacher expertise scale based on the qualitative data. The instrument was adapted by reference to the existing Scale of Occupational Expertise (Van der Heijden, 2003; Evers et al., 2011) and the Generalized Expertise Measure (Germain and Tejeda, 2012). This scale had 19 items, all of which were designed according to a 5-point Likert scale format, with 1 representing “strongly agree” and 5 representing “strongly disagree.” The dimension of knowledge structure (Cronbach’s alpha = 0.834) had 4 items (e.g., “I am able to master the subject knowledge and gain a deep understanding of it”). The dimension of teaching ability (Cronbach’s alpha = 0.949) had 9 items (e.g., “I pay close attention to students’ responses and am able to arrive at a correct judgment based on their language and body language”). The dimension of professional developmental agency (Cronbach’s alpha = 0.850) had 6 items (e.g., “No matter how busy I am, I always find time to participate in training activities”).

### 3.3. Data analysis

To develop a theoretical construct for teacher expertise and identify its components, grounded theory analysis was applied to conceptualize the coding scheme of teacher expertise. The qualitative phase resulted in themes that were used to create items for the quantitative survey. To validate the construct for teacher expertise developed in the qualitative phase, Confirmative factor analysis, Kruskal-Wallis test, and Mann-Whitney test were used to evaluate the construct validation and discriminant validation.

#### 3.3.1. Qualitative analysis

To develop a construct for teacher expertise and identify its components, the study analyzed 621 critical incident stories. These were transcribed into 300,000 words, with the longest story being 1,503 words long and the shortest 220 words long. The study adopted a grounded theory approach (Strauss and Corbin, 1998) to develop a coding scheme illuminating the characteristics of participants’ expertise. Open coding and axial coding were used to identify concepts, categories, or codes. The transcripts were read and analyzed independently by two researchers, and differences were discussed and reconciled to identify common themes. An iterative process using various coding strategies was conducted to define each code category, along with the corresponding codes and subcodes, within each main code dimension. Inductively deduced code categories, codes, and subcodes were critically assessed for their resemblance to existing concepts and codes in the extant literature. The process continued until the analysis reached the point of theoretical saturation (Carmichael and Cunningham, 2017). To ensure the reliability of the data analysis and trustworthiness of the findings, we took the following steps: (1) a triangulation between the different data sets and the existing literature on teacher expertise was conducted (Creswell and Poth, 2016); (2) member checking was conducted by inviting the teachers to read and comment on the preliminary analyses (Erlandson et al., 1993); and (3) rigorous discussions were held between the researchers and critical challenges were made, which facilitated the interpretation of the data. The intercoder reliability was 95%, which is in accordance with the standard of 80% (Saldaña, 2021).

#### 3.3.2. Quantitative analysis

To verify the validity of the construct for teacher expertise and its discriminant validity, the teacher expertise survey was analyzed using SPSS 17.0 and AMOS 17.0. Concerning the construct validity of the teacher expertise scale, SPSS 17.0 was used to analyze the descriptive statistics and calculate the correlation of the variables. AMOS 17.0 was used to conduct the confirmatory factor analysis. CFA can specify the number of factors required in the data and determine which measured variable is related to which latent variable. The optimal model is based on the fit indexes of χ^2^/df (< 3.0), GFI (> 0.90), CFI (> 0.90), RMSEA (< 0.08), and SRMR (< 0.08). Concerning the discriminant validity of the teacher expertise scale, the study compared the goodness-of-fit of models ranging from three-factor to single-factor models, and then compared three groups of teachers using Kruskal-Wallis test and Mann-Whitney test.

## 4. Results

### 4.1. The construct for teacher expertise and its components

A detailed description of the coding scheme, code dimensions, code categories, codes, percentages of occurring categories and codes, and an example of coded verbalization is given below.

**FIGURE 1:**
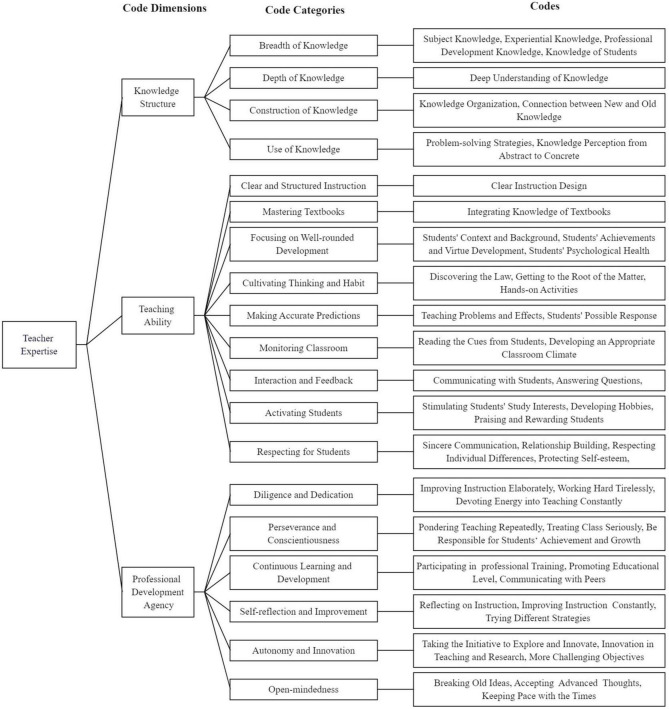
An overview of the code tree presenting all code dimensions, code categories, and codes included in the coding scheme.

Figure 1 shows the code tree, including all components of the coding scheme for clarifying teacher expertise. It presents the three code dimensions (i.e., knowledge structure, teaching ability, professional development agency) and their subdivision into code categories and codes.

Table 6 provides per code dimension descriptions and examples of the code categories and codes. In addition, the percentages of category and code occurrences within code dimensions are provided to illustrate their distribution throughout teacher expertise. The three code dimensions illustrate the structural and content components of teacher expertise.

**TABLE 6:** Coding scheme: Teacher expertise.

Code categories	Description	Example	% of occurrence
**Code dimension 1 knowledge structure**			
Breadth of knowledge	Extensive knowledge related to the subject being taught	Knowledge is not limited to the subject being taught but should be expanded as far as possible	100%
Depth of knowledge	Being proficient in subject knowledge	The subject knowledge taught to students is accurate and in-depth	23.5%
Construction of knowledge	Knowledge is interconnected into an integrated knowledge system and structure	To establish a knowledge system and knowledge network based on teaching experiences	9.8%
Use of knowledge	Knowledge is used effectively to solve problems in teaching	Exercise is placed in the context of a specific life situation	24.5%
**Code dimension 2 teaching ability**			
Clear and structured instruction	Being clear about teaching content, objectives, tasks, methods, and means	An excellent teacher has better methods and techniques of teaching	19.6%
Mastering textbooks	Accurately mastering the textbooks and knowledge of the subject being taught	An excellent teacher can explain the most complex knowledge in the textbook in simple terms and taking an easy-to-understand approach	9.8%
Focusing on students’ well-rounded development	Focusing on students’ achievement, together with views on life and values permeating into teaching	Cultivating students’ group consciousness and honesty, which is good for their achievement	10.7%
Cultivating students’ thinking and habit	Cultivating students’ thinking abilities and behavioral habits through teaching	Students are put into groups to share and display mind maps in class	29.4%
Making accurate predictions	Predicting possible problems and effects in teaching	Taking students’ possible responses into account in preparing lessons	8.8%
Monitoring class situations	Being sensitive to students’ responses to class situations and developing an appropriate learning culture	Creating an active classroom climate through communication and sharing among students	33.3%
Interaction and feedback	Interacting with students and providing them with in-time feedback	Making sure that while the teacher is speaking that the students grasp the key words, connect them with the context, make comparisons, and use other learning methods by giving timely affirmations and strengthening learning behaviors	63.7%
Activating students	Stimulating students’ interest and arousing their enthusiasm in learning	Stimulating students’ interest in learning by sharing stories and interesting news	31.6%
Respecting for students	Caring about students and respecting their performance and ideas	Understanding that teaching is based on rapport with the students and trying to establish trust-based relationships with them	15.7%
**Code dimension 3 professional development agency**			
Diligence and dedication	Working hard tirelessly and dedicating time and energy to self-improvement	I repeatedly invite my colleagues and leaders to listen to my courses and improve the quality of my teaching	55%
Perseverance and conscientiousness	Having a strong sense of conscientiousness, perseverance, and of high requirements for teaching	I take 2 weeks to go over my curriculum design over and over again until the optimal one is determined	16.7%
Learning and development	Participating in various learning activities	I have access to high-quality courses and excellent teachers through various channels	15.7%
Self-reflection and improvement	Reflecting and evaluating one’s teaching practice, taking notes, and making adjustment	The students’ level of achievement is not good. This is because my course is not clear enough, and there is not enough homework	51.0%
Autonomy and innovation	Thinking independently and solving teaching problems creatively	I expand the subject resources that are not in the textbook	12.3%
Open-mindedness	Being open to new ideas and new things	We should keep up with the pace of development of the times and not stick to conventions	20.6%

### 4.2. The validity of the construct for teacher expertise

First, we verify the construct validity of teacher expertise. The descriptive statistics, correlations, and construct reliability of the teacher expertise scale are shown in Table 7. The Cronbach’s alpha for each scale was between 0.818 and 0.881, illustrating reliable internal consistency.

**TABLE 7:** Correlation of each factor and total score of teacher expertise.

		Mean	SD	1	2	3	Composite reliability
1	Knowledge structure	4.05	0.70	(0.88)			0.932
2	Teaching ability	4.21	0.66	0.78**	(0.86)		0.962
3	Professional development agency	4.23	0.62	0.69**	0.75**	(0.82)	0.924

**p < 0.01, internal reliability values for the constructs are shown in parentheses on the diagonal.

As for the construct validity, as shown in Table 8, the fit indices of the three-factor model were good, χ^2^/df = 3.16, CFI = 0.95 > 0.90, GFI = 0.90, RMSEA = 0.078 < 0.08, SRMR = 0.039 < 0.08, illustrating a valid construct for teacher expertise.

**TABLE 8:** Fit indices of three-factor structural model of teacher expertise.

Model	χ^2^	df	χ^2^/df	GFI	CFI	RMSEA	SRMR
Three-factor model	451.78	143	3.16	0.905	0.95	0.078	0.039

**FIGURE 2:**
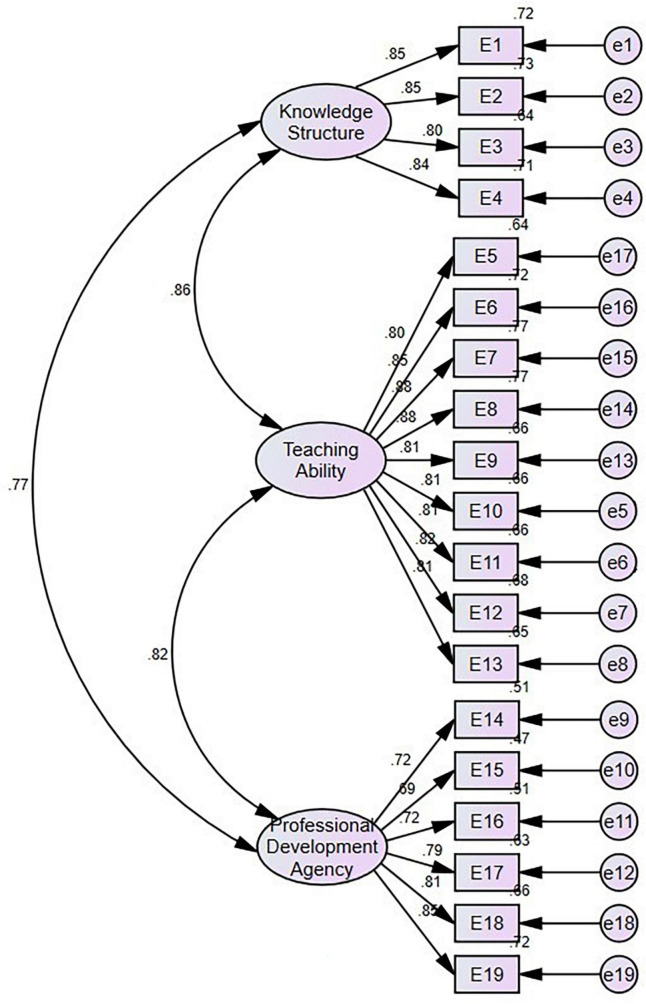
Confirmatory factor analysis of the three-factor model of teacher expertise.

As shown in Figure 2, the factor loading of each observed variable reached more than 0.69, and the pairwise correlation coefficients among the three factors were between 0.77 and 0.86. Therefore, the three-factor construct for teacher expertise has good construct validity.

Second, we verify the discriminant validity of teacher expertise. we compared the goodness-of-fit of models ranging from the three-factor to the single-factor model. The results presented in Table 9 show that the three-factor model fit the data best (χ^2^/df = 3.16; GFI = 0.90; CFI = 0.95; RMSEA = 0.078; SRMR = 0.039), indicating that knowledge structure, teaching ability, and professional development agency were three significantly different dimensions of teacher expertise.

**TABLE 9:** Results of the discriminant validity test and common method variance test.

Model	χ^2^/df	CFI	GFI	RMSEA	SRMR
3-factor (KS; TA; PDA)	3.16	0.95	0.90	0.078	0.039
2-factor (KS; TA + PDA)	7.45	0.84	0.72	0.135	0.063
2-factor (KS + PDA; TA)	7.18	0.85	0.72	0.132	0.058
2-factor (KS + TA; PDA)	6.75	0.86	0.74	0.127	0.053
1-factor (KS + TA + PDA)	8.70	0.81	0.68	0.147	0.068

KS, knowledge structure; TA, teaching ability; PDA, professional development agency.

To further examine the validity of the teacher expertise construct for differentiating between expert and non-expert teachers according to their levels of teacher expertise, we compared three groups of teachers. The first group (*N* = 138) was composed of novice teachers who had been teaching for less than 4 years and had not won any honors. The second group (*N* = 2,148) was composed of experienced teachers who had been teaching for 4 to 10 years and had received honors at the below-district level. The third group (*N* = 226) comprised expert teachers with over 10 years of teaching experience who had received honors at the district level or above.

First, we tested whether the data of teacher expertise and its three dimensions fitted as normal distribution. The result in Table 10 showed that for novice, experienced, and expert teachers, teacher expertise and its three dimensions did not fit as normal distribution. Therefore, Kruskal-Wallis test was employed to examine whether a statistically significant difference exist between the means of teacher expertise and its three dimensions among different groups of teachers. The result in Table 11 indicated that there were significant differences among novice, experienced, and expert teachers in terms of teacher expertise, teaching ability, and professional development agency. However, as for knowledge structure, no significant difference was observed.

**TABLE 10:** Test of normal distribution.

	Teacher expertise	Knowledge structure	Teaching ability	Professional development agency
Novice teacher (*N* = 138)	0.049	0.001	0.002	0.002
Experienced teacher (*N* = 214)	0.001	0.001	0.001	0.001
Expert teacher (*N* = 226)	0.002	0.001	0.001	0.001

**TABLE 11:** Kruskal-Wallis test of teacher expertise between novice, experienced, and expert teachers.

	χ^2^	df	*P*
Teacher expertise	12.220	2	0.002
Knowledge structure	4.547	2	0.103
Teaching ability	14.164	2	0.001
Professional development agency	12.220	2	0.000

To take a closer insight into the concrete difference of teacher expertise and its three dimensions among different groups of teachers, we conducted three Mann-Whitney tests between novice and experienced, between experienced and expert, and between novice and expert teachers. The result in Table 12 showed that the differences in teacher expertise between novice and expert teachers were significant (*p* < 0.01), as were those between experienced and expert teachers (*p* < 0.01). Specifically, in terms of the three dimensions of teacher expertise, novice and experienced teachers had significantly different levels of teaching ability and professional development agency from expert teachers (*p* < 0.05), whereas there were no significant differences in knowledge structure for the three groups. The results verified that the construct for teacher expertise can differentiate expert teachers from experienced and novice teachers in terms of their levels of teaching ability and professional development agency.

**TABLE 12:** Means, SDs, and Mann-Whitney test of teacher expertise between novice, experienced, and expert teachers.

		Novice teacher (*N* = 138)	Experienced teacher (*N* = 214)	Expert teacher (*N* = 226)		Novice vs. experienced	Experienced vs. expert	Novice vs. expert
Teacher expertise	M	4.059	4.056	4.205	Z	-0.152	-3.089	-2.804
	SD	0.523	0.581	0.612	P	0.879	0.002	0.005
Knowledge structure	M	3.992	3.979	4.082	Z	-0.330	-1.940	-1.605
	SD	0.619	0.672	0.713	P	0.742	0.052	0.108
Teaching ability	M	4.042	4.135	4.261	Z	-1.381	-2.439	-3.667
	SD	0.617	0.614	0.652	P	0.167	0.015	0.000
Professional development agency	M	4.145	4.053	4.273	Z	-1.341	-4.123	-2.606
	SD	0.548	0.622	0.631	P	0.180	0.000	0.009

## 5. Discussion

The objective of this study was to develop a construct for teacher expertise in the Chinese context, identify its components, and verify the validity of the construct. The qualitative results demonstrated that teacher expertise is a complex, multidimensional, and adaptive construct in which the cognitive and affective components may interact with and support each other. The quantitative results confirmed that the construct for teacher expertise has good construct validity and should be a reliable tool for discriminating expert teachers from experienced and novice teachers.

### 5.1. The construct for teacher expertise and its components in the Chinese context

On the basis of the qualitative results, teacher expertise can be understood to refer to the sum of a teacher’s personal characteristics that enable them to effectively solve teaching problems based on personal knowledge, professional experience, reflection on practice, and innovative activities. First, knowledge structure refers to teachers’ knowledge systems, including breadth of knowledge, depth of knowledge, construction of knowledge, and use of knowledge. Knowledge has come to be seen as a major component of teacher expertise, underpinning how teachers help students learn science, as well as develop their ability to inquire. Second, teaching ability refers to teachers’ problem-solving ability in response to the dynamic and complex characteristics of the teaching process, including clear and structured instruction, mastering textbooks, focusing on students’ well-rounded development, cultivating students’ thinking and habits, making accurate predictions, monitoring the classroom, interacting with students and providing them with feedback, activating students, and respecting students. Teaching ability is the core component of teacher expertise, which is not a fixed storehouse of facts and ideas but a source and creator of the knowledge and skills needed for instruction. Third, professional development agency refers to the affective and motivational components of teachers’ professional development, including diligence and dedication, perseverance and conscientiousness, continuous learning and development, self-reflection and improvement, autonomy and innovation, and open-mindedness. The pursuit of professional development is a significant characteristic of expert teachers (Sternberg and Horvath, 1995; Smith and Strahan, 2004; Silver et al., 2019). The role of agency in developing teachers’ adaptive expertise is emphasized (Anthony et al., 2015). Teachers who have agency are aware of the need for professional development (Christiansen et al., 2018) and can promote learning and facilitate professional development (Lee, 2010).

Our findings are consistent with the notion that teacher expertise is not merely a state indicating what a teacher knows (Shulman, 1986) or how they behave as an expert on teaching (Berliner, 2004), but it is also a process manifesting how the teacher interacts with the context and engages in learning for knowledge growth and optimal behaviors (Peercy et al., 2015). These results are also consistent with our previous studies on the components of expertise among mathematics teachers in primary schools (Cai et al., 2015, 2016). This confirms the rationality of the components of teacher expertise in our previous studies.

### 5.2. The validity of the construct for teacher expertise

The quantitative results proved that the theoretical construct for teacher expertise developed in the qualitative phase was a valid and reliable instrument to differentiate between expert and non-expert teachers. However, knowledge structure did not differentiate expertise sufficiently. Teaching ability and professional development agency could identify and develop expertise. The results confirm that teacher expertise is not about the extent of an expert’s knowledge. It is better understood as a process of doing. Teachers’ professional development agency is important to the practice of expert teachers. In this study, expert teachers have a strong sense of responsibility and morality, which can be seen in their diligence, dedication, perseverance, and conscientiousness. From this perspective, the development of teacher expertise depends on the cultivation of the virtue of morality, which is a philosophical concept meaning rightness or goodness. This is due to the Confucian cultural context, which emphasizes teachers’ virtue and social responsibility. By comparison, in studies from the Western context, the development of teacher expertise depends on meeting teachers’ achievement motives, which is a psychological concept that originates from needs and emphasizes the meeting of individual needs. This is due to the individualistic culture in the West and the instrumental value placed on teaching, which emphasizes individual value and development and meeting one’s own individual needs.

## 6. Implications

### 6.1. Theoretical implications

This study systematically and comprehensively examines a construct for teacher expertise in the Chinese context and its components and provides much-needed empirical support for the construct validity and discriminant validity of teacher expertise using mixed methods. Thus, this study goes beyond previous studies on routine expertise that focused on only a limited set of expert teachers’ characteristics or personality traits. Apart from the cognitive dimensions of teacher expertise, the affective components have yet to be emphasized. Furthermore, the existing literature is largely based on the Western context. This study proves that there are cultural differences in ideas about what constitutes an expert teacher. This study contributes to the research on teacher expertise by establishing an overarching framework that can be used as a springboard for further research in different cultural contexts. Most importantly, this study highlights the role of agency in developing teacher expertise. Obtaining professional development agency is an important signal of the development of expertise. Professional development agency is the most important component differentiating expert teachers from experienced or novice ones. Prior studies on adaptive expertise have underlined the motivational components that increase expertise, particularly adaptability. In addition to the motivational components, this study emphasized the virtue of morality as an important component of professional development agency. Future research should pay more attention to the mechanisms that motivate teachers’ professional development agency.

### 6.2. Practical implications

This study provides a reliable tool for identifying and developing teacher expertise and helping teachers acquire expertise. Teacher expertise is usually assessed in terms of teachers’ knowledge and teaching skills. This focus on the external performance of teaching does not address the internal motivations that shape the concepts and actions taken by teachers. However, this study concluded that knowledge structure is not significant in differentiating novice and expert teachers. Therefore, the adoption of the novice-to-expert continuum in the form of graded assessment grids cannot sufficiently differentiate expertise. Teaching is recognized as a practice with values, emotions, and intentions that involves social and relational processes with those being taught. In other words, teaching is a complex, social, and relational practice. In this way, this study recognizes teacher expertise as a multidimensional construct and emphasizes teachers’ agency as being important to the practice of expert teachers. The findings of this study add to the understanding of teacher expertise by addressing how and why expert teachers know how to act in teaching. This contrasts with the more general graded descriptors of what teachers should know or be able to do. Attempting to compel teachers to acquire expertise is problematic because expertise is not an action that can be mimicked without an appreciation of the underlying principles and reasons for action. This study may provide useful implications for facilitating teachers’ continuous learning and growth of expertise.

## 7. Limitations and further research

First, this study developed a theoretical model for teacher expertise and examined its components using critical incident interviews and grounded theory methods. However, self-reported critical incidents may be falsified, and the situations and moments chosen may not be fully representative of what is to be evaluated. In addition, personal experiences may be difficult to talk about for privacy reasons, which can limit the answers given. Future research may investigate the effectiveness of this method compared to other methods, such as observation, laboratory, narrative inquiry, and ethnography. Sociocultural approaches that stress the situated nature of knowledge, learning, and action could be used to more widely explore concepts about teacher expertise developed from teachers’ motivations and in terms of their actions.

Second, the teachers who participated in this study were not selected based on their individual ideas, and teachers’ views on expertise can differ across individuals. The differences in teacher expertise should be compared across different levels, phases, and subjects. Future research could use additional selection criteria for participants to specify the coding scheme and enrich the understanding of teacher expertise.

Third, expertise in authentic contexts should be studied. Expertise develops as long as individuals are exposed to situations in which they have to overcome the restrictions of their earlier stages. Context plays a major role in directing or affecting the development of teacher expertise. Future research could compare teacher expertise across different specific contexts.

Fourth, future research could explore the interrelationships between knowledge, skills, professional development agency, and expertise. In addition, given the importance of agency in teaching, future research that investigates affective, cognitive, and motivational mechanisms merits further attention.

## Data availability statement

The datasets presented in this article are not readily available because they contain participants’ personal information. Requests to access the datasets should be directed to YC, caiyonghong@bnu.edu.cn.

## Author contributions

YC: conceptualization, methodology, investigation, writing—original draft, writing—review and editing, project administration, and funding acquisition. YL: writing—formal analysis, data curation, writing—original draft, writing—review and editing, and visualization. RT: formal analysis, data curation, writing—review and editing, and visualization. All authors contributed to the article and approved the submitted version.
